# Protein Lipidation As a Regulator of Apoptotic Calcium Release: Relevance to Cancer

**DOI:** 10.3389/fonc.2017.00138

**Published:** 2017-06-29

**Authors:** Jessica J. Chen, Darren Boehning

**Affiliations:** ^1^Department of Biochemistry and Molecular Biology, McGovern Medical School, UTHealth, Houston, TX, United States

**Keywords:** calcium, apoptosis, lipidation, Fas, cancer, inositol phosphates, kinases, statins

## Abstract

Calcium is a critical regulator of cell death pathways. One of the most proximal events leading to cell death is activation of plasma membrane and endoplasmic reticulum-resident calcium channels. A large body of evidence indicates that defects in this pathway contribute to cancer development. Although we have a thorough understanding of how downstream elevations in cytosolic and mitochondrial calcium contribute to cell death, it is much less clear how calcium channels are activated upstream of the apoptotic stimulus. Recently, it has been shown that protein lipidation is a potent regulator of apoptotic signaling. Although classically thought of as a static modification, rapid and reversible protein acylation has emerged as a new signaling paradigm relevant to many pathways, including calcium release and cell death. In this review, we will discuss the role of protein lipidation in regulating apoptotic calcium signaling with direct therapeutic relevance to cancer.

## Introduction

Apoptotic cell death is important for embryonic development and tissue homeostasis, and dysfunction of this pathway can contribute to various disease states, including cancer. The intrinsic apoptosis pathway is activated by cellular stress and leads to Bcl-2 protein activation, mitochondrial membrane permeabilization, and release of proapoptotic proteins (1, 2). In addition to their roles in mitochondrial membrane permeabilization, Bcl-2 family proteins are also essential and direct regulators of intracellular calcium during apoptosis by binding to a surprising variety of channels, pumps, and exchangers (3–11). Elevated cytosolic calcium then contributes to the apoptotic program in a multitude of ways including mitochondrial permeabilization and further activation of proapoptotic Bcl-2 proteins (12).

The extrinsic apoptosis pathway is activated by ligand binding to death receptors of the tumor necrosis factor-α (TNFα) superfamily. Ligand binding to the death receptor results in the activation of the initiator caspases 8 and 10 (13–15). Caspase 8/10 can directly cleave and activate effector caspases and/or cleave the proapoptotic Bcl-2 family protein Bid. Truncated Bid leads to mitochondrial permeabilization and release of proapoptotic proteins (16). Cell which do not engage the mitochondrial pathway are called “type I” cells, and those which lead to mitochondrial permeabilization are called “type II” cells (17). Thus, in type II cells the extrinsic pathway converges with the intrinsic pathway at the mitochondria. Calcium also contributes to the progression of the extrinsic pathway (18–21); however, this is less well understood.

Recently, it has been found that multiple proteins which regulate apoptotic calcium release in both the intrinsic and extrinsic pathways are subject to lipidation. Protein lipidation is the cotranslational or posttranslational covalent addition of a variety of lipids, including fatty acids, isoprenoids, and cholesterol, to target proteins. Such modifications regulate protein localization and function in many signaling processes. Recent advances in detecting lipidated proteins by proteomic and targeted approaches have revealed that lipidation of signaling proteins is essential for regulating a wide variety of signaling pathways. Stimulus-dependent lipidation of the apoptotic machinery is likely a central regulator of cell death, and defects in this pathway may be contributing factors in cancer development. In this review, we will discuss how protein lipidation plays an essential role in apoptotic signaling and the relevance to cancer therapeutics.

## Types of Lipidation

Lipidation can be categorized into two types based on the location of the modified proteins: those that are modified in the ER lumen and secreted and those that are modified in the cytoplasm or on the cytoplasmic face of membrane (22). The former type includes glycosylphosphatidylinositol (GPI) anchor and cholesterylation, and the latter includes N-myristoylation, acylation, and prenylation.

Glycosylphosphatidylinositol anchor was first discovered in the parasite *Trypanosoma brucei* where the highly expressed variant surface glycoprotein is anchored to the cell surface *via* a glycolipid containing phosphatidylinositol (23–25). Since then, many proteins in mammals and lower eukaryotes such as protozoa have been shown to contain GPI anchors with enormous structural variety, most of which include an ethanolamine attached to the carboxyl terminus of the protein, a glycan core, inositol, and lipid moieties (26–28). GPI-anchored peptides often include a cleavable N-terminal signal sequence, which directs the peptide to ER lumen, and a hydrophobic C-terminal sequence that is cleaved at the time of GPI anchor addition (27, 29, 30). GPI anchors facilitate tethering of proteins to the extracellular face of the plasma membrane and are important for many cellular functions, including adhesion, membrane trafficking, and immune system signaling (31–33).

Cholesterylation is a characteristic of the mammalian Hedgehog family, which are secreted signaling proteins that regulate embryonic patterning of many tissues and structures (34, 35). The Hedgehog protein undergoes an autocatalytic processing that internally cleaves between the conserved Gly^257^ and Cys^258^ at the GCF motif and yields a ~20 kDa N-terminal signaling domain and a ~25 kDa C-terminal catalytic domain (36, 37). The N-terminal domain receives a cholesterol moiety and is active in signaling (35, 37, 38). Interestingly, multiple studies have detected other potentially cholesterylated proteins (35, 39). However, the identification of these potential cholesterylation targets remains to be elucidated.

N-myristoylation is the attachment of the 14-carbon myristic acid to an N-terminal Gly residue *via* an amide bond (40). It was first identified as a blocking group that prevents Edman degradation on the N-terminus of the catalytic subunit of cyclic AMP-dependent protein kinase and the calcium-binding β-subunit of calcineurin (41, 42). Many other proteins regulating key signaling pathways, including the Src family non-receptor protein tyrosine kinases (43, 44) and G_α_ proteins (45, 46) were shown to be myristoylated. These proteins contain the N-terminal sequence Met–Gly– and often have a Ser/Thr/Cys at position 6 (40, 47). Myristoylation can happen cotranslationally following the removal of the initiator methionine residue (48). Although myristoylation is required for membrane targeting of many proteins, it is not sufficient for stable membrane anchoring due to its weak hydrophobic nature and often needs subsequent lipid modifications (49–51). Additionally, myristoylation can also happen posttranslationally during apoptosis following the caspase cleavage of substrate proteins that exposes an internal glycine (52–55). Many apoptotic proteins, including Bid, gelsolin, and p21-activated kinase 2, require posttranslational myristoylation following caspase cleavage for proper subcellular localization and subsequent functions (52–54).

Acylation is the addition of various fatty acids, such as palmitic acid, oleic acid, and stearic acid, on different amino acid residues (56–58). One of the best studied types of acylation is S-palmitoylation, which is characterized by the reversible addition of the 16-carbon saturated palmitic acid to Cys residues *via* labile thioester bonds (58, 59). Despite the presence of multiple algorithms to predict palmitoylation sites, there is no validated consensus sequence for palmitoylation (60–62). One key aspect of palmitoylation is that its reversibility allows for a palmitoylation and depalmitoylation cycle that regulates the posttranslational trafficking and functions of target proteins, such as H- and N-Ras (63). Palmitoylation can also occur on large transmembrane proteins, including ion channels and G protein-coupled receptors (64–68). The roles of transmembrane protein palmitoylation include regulation of channel maturation/quality control and association with lipid rafts (69–72).

Prenylation is the addition of the 15-carbon farnesyl or the 20-carbon geranylgeranyl isoprenoid lipid on cysteine residues *via* stable thioether bonds (73, 74). It requires a C-terminal CAAX motif, where C is a cysteine, A is aliphatic amino acids, and X can be any amino acid. Prenylation at the CAAX motif is found in many proteins, including mammalian Ras proteins (75, 76). In addition to its role in membrane association, prenylation can also regulate protein–protein interaction and subcellular distribution of the modified targets (77, 78).

## Enzymatic Regulators of Protein Lipidation

Protein lipidation is catalyzed by specific enzymatic regulators crucial for the addition (and removal in the case of S-acylation) of the lipid moieties. The GPI precursor, formed in ER lumen, is transferred to target proteins by GPI transamidase, a membrane-bound multi-subunit enzyme (79–82). GPI transamidase cleaves the C-terminal signal peptide of the target proteins, and forms an enzyme-substrate intermediate, allowing the nucleophilic attack by the terminal amino group of preformed GPI (83). On the other hand, cholesterylation of the N-terminal signaling domain of Hedgehog seems to be only dependent on the presence of the C-terminal catalytic domain, suggesting that this process is autocatalytic (35).

N-myristoylation is catalyzed by *N*-myristoyltransferases (NMTs) (84–86). NMTs bind first to myristoyl-CoA and then to the peptide, followed by a direct nucleophilic addition–elimination reaction and subsequent release of CoA and the myristoylated peptide (87). Studies in various tissues and cell types have shown that the enzymatic activity of NMTs is predominantly distributed in the cytosolic fraction (88–91). Some studies have shown that low levels of myristoyl-CoA may be rate limiting for NMT activity (92, 93). However, the transcriptional upregulation of NMT under pathological conditions suggests that this might not always be the case (94).

Protein S-acylation is regulated by palmitoyl acyltransferases (PATs) that catalyze lipid attachment to cysteine residues and acyl-protein thioesterases (APTs), which remove them. There are 23 PATs in mammals, all of which share a common DHHC (Asp–His–His–Cys) motif within a cysteine-rich domain (95, 96). PATs are polytopic membrane proteins that are localized to distinct subcellular compartments, primarily the Golgi apparatus and the plasma membrane (97). Some DHHC enzymes show preference for certain types of proteins (i.e., transmembrane proteins), and in some cases the same substrates can be palmitoylated by multiple DHHC enzymes (95, 98–100). Compared to the large amount of studies on PATs, thioesterases are relatively poorly characterized. Two protein palmitoyl thioesterases (PPT1/2) and two APTs (APT1/2) have been identified (101–104). PPTs predominantly localize to the lysosomal lumen and are involved in depalmitoylation during protein degradation, whereas APTs have cytosolic localization and are shown to depalmitoylate and recycle signaling proteins such as Ras and growth associate protein (GAP-43) from the plasma membrane back to Golgi (102–105). Very recently, two independent groups found that the α/β hydrolase fold (ABHD) family of serine hydrolases is potent depalmitoylating enzymes for select substrates, including PSD-95 and N-Ras (106, 107).

Prenylation is catalyzed by the enzymes farnesyltransferase (FTase), geranylgeranyltransferase I (GGTase 1), and Rab geranylgeranyltransferase (GGTase 2) (108–110). The prenylating enzymes localize to the cytosol and conjugate isoprenoids generated from mevalonate/HMG-CoA reductase pathway to target proteins. Specifically, the isoprenoids farnesyl and geranylgeranyl are transferred to a C-terminal CAAX motif on target proteins. Unlike FTase and GGTase 1, geranylgeranyl transfer by GGTase 2 requires the co-factor REP (Rab escort protein) (111). GGTase 1 and FTase generally have high specificity for the protein targets, depending on the X residue (112–114). However, they can act on each other’s substrates. One example is that K-Ras and N-Ras, usually targets of FTase, can be geranylgeranylated in Ras-mutant human cancer cells treated with FTase inhibitors (115, 116). Removal of the –AAX tripeptide and methylation of the prenyl-cysteine, catalyzed by the ER membrane proteins RCE1 and ICMT, respectively, are two post-prenylation steps required for maturation of prenylated proteins (117–119).

## Lipidation and Apoptotic Calcium Release

As noted in the Section “Introduction,” calcium regulates many cellular processes and plays a prominent role in cell death signaling. Both intrinsic (12, 120–122) and extrinsic (18–21, 123) apoptotic stimuli lead to cytosolic, nuclear, and mitochondrial calcium elevations, which contribute to the execution of the apoptotic program. It is well known that many proteins that regulate cytosolic calcium and apoptotic calcium release are also subject to lipidation including pumps (124), exchangers (125), channels (126), and regulatory proteins (127). Perhaps best studied is protein palmitoylation due to the proliferation of proteomic studies using acyl-biotin exchange (ABE) to identify fatty-acylated proteins (128). Proteins are often assumed to be palmitoylated in ABE experiments, but clearly other lipids may also be conjugated with a thioester bond to target protein cysteine residues (129).

In order to understand how palmitoylation contributes to apoptotic calcium signaling, it is worthwhile to considering the kinetics of the enzymatic machinery. In many proteins which do not have a transmembrane domain, N-terminal myristoylation precedes palmitoylation (130). Over 15 years ago, it was shown that β-adrenergic stimulation resulted in rapid palmitoylation (130, 131) or depalmitoylation (132) of G_αs_. The model was based upon availability of free G_αs_: dissociation from the βγ subunits allowed putative palmitoylating and depalmitoylation enzymes access to the protein. Under this model, regulated palmitoylation and depalmitoylation cycles would be restricted to proteins which, under physiologic conditions, had regulated exposure of potential palmitoylation sites. Indeed, G_αs_ is one of only a very select few proteins in which direct palmitoylation within minutes of cellular stimulation has been conclusively determined [although other proteins such as PSD-95, eNOS, and Ras clearly have much higher turnover of palmitoyl groups in response to various stimuli, suggesting rapid cycling of lipid (133)].

**Figure 1:**
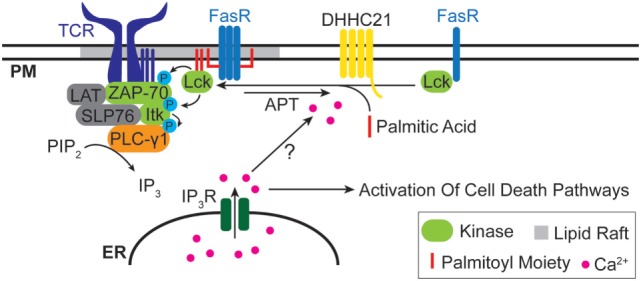
Lipidation and Fas death receptor signaling. The Src kinase Lck is rapidly palmitoylated upon Fas stimulation and partitioned into lipid rafts, where it interacts with the T cell receptor (TCR) complex and leads to downstream apoptotic calcium release. The plasma membrane-localized palmitoyl acyltransferases DHHC21 is essential for Lck palmitoylation. The identity of the depalmitoylating enzyme(s) for Lck is unclear.

Many proteins associated with cell death signaling are modified by lipids. Our group (18) and others (134–137) have investigated the role of palmitoylation in regulating death receptor signaling. We found that components of the T cell receptor (TCR) complex, such as Lck, Zap-70, PLC-γ1 and other TCR components were required for apoptotic calcium release in T cells after engagement of the Fas receptor with Fas ligand (19). The Src kinase Lck is myristoylated and doubly palmitoylated on the N-terminus, and this regulates plasma membrane localization and partitioning into lipid rafts. It is known that the Fas macromolecular complex assembles and signals in lipid rafts (138), so we asked whether Fas stimulation resulted in rapid palmitoylation of Lck. Fas stimulation resulted in a rapid increase in *de novo* palmitoylation of Lck detectable within minutes of Fas receptor engagement (18). Unexpectedly, the lipid moiety was removed from Lck almost as quickly, and Lck palmitoylation was almost undetectable by 30 min. These kinetics closely matched the phosphorylation and de-phosphorylation of canonical TCR components, such as Zap-70 and PLC-γ1 (18). These findings strongly suggest that the enzymatic mechanisms controlling stimulus-dependent protein palmitoylation and depalmitoylation likely are directly activated by components of the Fas signaling pathway. In the case of Fas signaling, the plasma membrane-localized DHHC21 protein is essential (18). Presumably Fas stimulation rapidly activates DHHC21 and a yet unidentified acyl-protein thioesterase to regulate Lck lipidation levels (Figure 1). How the activation of these enzymes occurs is unknown, but could possibly be regulated either directly or indirectly by calcium ions.

In addition to palmitoylation, there has been intense interest in how other lipid modifications are coupled to the cell signaling/cell death machinery. As mentioned above myristoylation of Src kinases regulates their localization to the plasma membrane, which would have implications for coupling to the apoptotic calcium release machinery. The Src-related kinase c-Abl is recruited to membranes *via* a myristoyl/phosphotyrosine switch (139, 140). The c-Abl kinase sequesters the myristoyl group in a hydrophobic pocket, a conformation which is essential for autoinhibition of the enzyme (140). Binding of phosphotyrosine ligands to the SH2 domain causes a conformational change which results in displacement of the myristoyl group from the pocket, activation of the kinase, and membrane localization. Thus, even though myristoylation is a stable modification, the regulated exposure of the lipid group allows stimulus-dependent regulation of the kinase. During cell death induced by calcium store depletion and subsequent ER stress, c-Abl translocates to mitochondrial membranes to stimulate cytochrome *c* release (141). It is important to note that the myristoyl switch is not conserved in all Src-related kinases. For example, Lck has bulky residues which occlude the myristoyl binding pocket, and combined with biochemical evidence (142, 143), suggest that a myristoyl switch does not operate in Lck.

Prenylation is a prominent modification of many disease relevant proteins. Small GTPases, such as Rab, Rho, and Ras, are arguably the best studied prenylated proteins; however, as mentioned above, many other proteins have also been shown to be prenylated including chaperones, kinases, enzymes, and receptors (110). How prenylation is linked to apoptotic calcium release has mostly been elucidated by studying the effects of HMG-CoA reductase inhibitors (more commonly known as statins). Many studies have shown that statins induce cell death of multiple cell types, and it is generally assumed to be due to decreased prenylation of GTPases such as Ras and Rho (144–147). We showed that simvastatin was a potent inducer of calcium-dependent apoptosis in human leiomyoma cells (148). The mechanism included inhibition of ERK signaling downstream of Ras and activation of plasma membrane L-type calcium channels *via* a still unknown mechanism. We were able to extend these finding to xenograft models (149) and showed in a nested case–control study that statin users had a lower risk of uterine fibroid tumors and related symptoms (150). Statins may also be effective for inhibiting the proliferation of many other tumor types including breast (151), ovarian (152), colon (153), and other cancers.

## Lipidation and Calcium-Dependent Tumor Progression

In addition to regulating cell death signaling, calcium is also a key factor in proliferative and pro-metastatic signaling. In many types of cancer, alterations in expression, localization, and functions of calcium pumps and channels are observed, resulting in ectopic calcium flux across the plasma membrane or intracellular organelles (154). Studies in prostate (155–157), colon (158), breast (159, 160), and ovarian (161) cancers demonstrated that multiple transient receptor potential (TRP) channels, a family of calcium permeable ion channels, are overexpressed and regulate proliferation in primary tumors. Additionally, TRP channels contribute to tumor cell migration by generating localized calcium signals that guide the direction of movement toward growth factors (162, 163). Another calcium-dependent pathway related to metastasis is the store-operated calcium entry (SOCE) mediated by ER calcium sensor stromal interaction molecule 1 and the plasma membrane calcium channel ORAI1. This pathway has been extensively studied in breast cancer, where it accelerates the turnover rate of focal adhesions by reorganizing the actin cytoskeleton in a Ras and Rac-dependent manner (164). The SOCE pathway is activated by G-protein coupled receptor-mediated activation of the phospholipase C-IP_3_R pathway, which results in calcium release from ER stores and contributes to metastasis by promoting actin assembly (165, 166).

**Figure 2:**
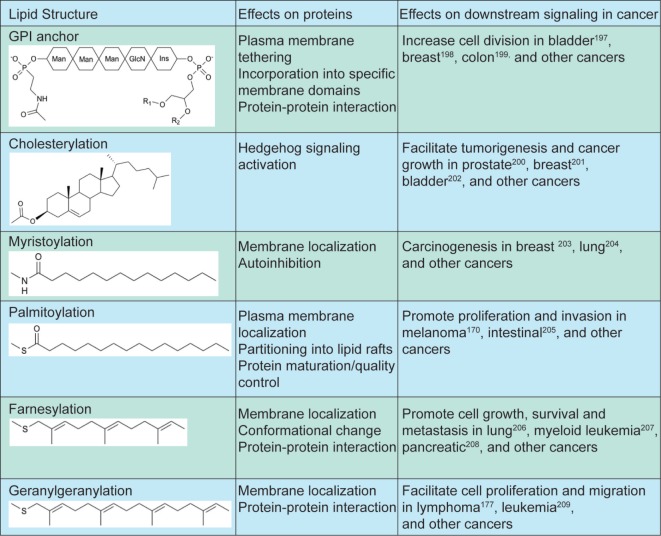
Lipidation structures and effects on downstream signaling in cancer. Increase GPI transamidase activity leads to increased cell proliferation in bladder (174), breast (175), and colon (176) cancers. Elevated hedgehog signaling is linked to sporadic tumorigenesis in prostate (177), breast (178), and bladder (179) cancers. Increased NMT activity and myristoylated Src kinases are linked to increased cell proliferation in breast (180), lung (181), and other cancers. Palmitoylation of signaling proteins in multiple pathways are linked to proliferation and invasion in melanoma (171), intestinal (182), and other cancers. Targeting farnesylation of Ras proteins slows down tumor progression in lung (183), leukemia (184), pancreatic (185), and other cancers. Geranylgeranylation of small GTPases facilitate cell proliferation and migration in lymphoma (186), leukemia (187), and other cancers.

Many proteins involved in the calcium-dependent proliferative and pro-metastatic pathways are regulated by lipidation (Figure 2). The Wnt signaling pathway is an extensively studied mediator of tumor progression. Immature Wnt proteins (with the exception of WntD) require N-glycosylation and lipidation, specifically palmitate/palmitoleic acylation on conserved C77/C93 and S209/S24 residues for proper secretion and subsequent recognition by Frizzled (Fzd) receptors (167–169). In addition to canonical β-catenin-dependent Wnt signaling, Wnt ligands such as Wnt5a bind Fzd receptors and activate PLC *via* G-proteins leading to IP_3_R-mediated increase in cytosolic calcium levels (170). Activation of the non-canonical Wnt/Ca^2+^ pathway has been implicated in multiple cancer types, including melanoma where it promotes invasion by initiating epithelial-to-mesenchymal transition (171, 172). Increasing *in vitro* data indicate that Wnt lipidation at the two sites is differentially regulated and activates distinct canonical versus non-canonical pathways (173), but their exact functions in different types of cancers remain unclear. Therefore, further characterization of Wnt lipidation and the mechanisms through which they regulate calcium-dependent proliferation and migration is necessary.

Another class of lipidated proteins that are involved in calcium-mediated cancer progression is the small GTPases, including Ras, Rho and Rac, which are known regulators of the calcium-dependent cytoskeletal rearrangement (164, 188). All three major mammalian isoforms of Ras (H-Ras, N-Ras and K-Ras4B) are farnesylated at the C-terminus (189). Ras farnesylation is required for membrane localization and activation of downstream pathways to induce tumorigenesis (190). Additionally, H-Ras and N-Ras are palmitoylated in the Golgi and subsequently localized to the plasma membrane, where it can be depalmitoylated and cycled back to the Golgi, leading to spatial and temporal control of Ras signaling (191). Rho and Rac are geranylgeranylated near the C-terminus and blocking geranylgeranylation leads to reduced cancer cell proliferation and migration (186, 192).

## Lipidation as a Drug Target in Cancer

Many oncogenic proteins require lipidation for proper function. Indeed, Ras is one of the most commonly mutated proteins in cancer (193). As such, the enzymes that mediate these modifications are excellent targets for drug development. Inhibitors of prenylation enzymes GGTase 1 and FTase are being developed to treat cancer. The GGTase 1 inhibitor PTX-200 (GGTI-2418) is being tested in clinical trials by Prescient Therapeutics for breast cancer and multiple myeloma; however, the Phase I trial data has not been published. Many more drugs have been developed which target FTases. There have been several clinical trials conducted with FTase inhibitors, such as lonafarnib, and tipifarnib (194); however, the results have been mostly disappointing (195). Interestingly, positive clinical responses were not correlated to Ras mutation status, suggesting that the drugs target other pathways or substrates (196). Drugs targeting the post-prenylation processing enzymes RCE1 and ICMT are in pre-clinical development (195, 197–199).

As mentioned above, many oncogenic proteins require palmitoylation for proper function, including Ras and Src proteins. There are very few pharmacological tools to target the DHHC enzymes; however, the irreversible lipid-based inhibitor 2-bromopalmitate (200) and several non-lipid reversible inhibitors (201) are widely used as research tools to probe the role of palmitoylation in biological processes. There are no drugs sufficiently well developed to initiate clinical trials, most likely due to the fact that selective DHHC enzyme inhibitors would likely need to be developed. As there are so many enzymes with potentially overlapping substrates, this seems to be a very daunting task. However, new targeted screening strategies for therapeutically relevant substrates, such as Ras, show great promise (202).

As mentioned above, statins are potent inhibitors of the mevalonate pathway and thus may be an attractive target for further development as anti-cancer agents. Indeed, several prospective and retrospective studies have shown that statins have activity against a wide variety of cancers (203–209). There are a multitude of prospective clinical trials currently underway to evaluate the potential of statins as anti-cancer therapeutics. Interestingly, the mechanism of action for inhibiting cancer progression by statins may reflect targeting of multiple pathways, including prenylation of oncogenic proteins and the production of hormones synthesized from cholesterol such as estrogen and testosterone which can drive tumor growth. Statins are one of the most widely prescribed drugs in the world with excellent safety and tolerability profiles (210). If statins prove to be efficacious as a cancer preventative or treatment, this would have the potential to revolutionize cancer care and survival.

## Author Contributions

The content of this review was conceived by DB and written with equal effort by JC and DB.

## Conflict of Interest Statement

The authors declare that this review was written in the absence of any commercial or financial relationships that could be construed as a potential conflict of interest.
